# Enhancing image quality in computed tomography angiography follow-ups after endovascular aneurysm repair: a comparative study of reconstruction techniques

**DOI:** 10.1186/s12880-024-01343-z

**Published:** 2024-07-01

**Authors:** Huasong Cai, Hairong Jiang, Dingxiang Xie, Zhiman Lai, Jiale Wu, Mingjie Chen, Zhiyun Yang, Rulin Xu, Shanmei Zeng, Hui Ma

**Affiliations:** 1 Department of Radiology, The First Affiliated Hospital, Sun Yat-sen University, No. 58, Zhongshan Er Road, Guangzhou, Guangdong Province 510080 People’s Republic of China; 2 Department of Radiology, Foresea Life Insurance Guangzhou General Hospital, No. 703, Xincheng Avenue, Zengcheng District, Guangzhou, Guangdong 511300 China; 3 Research Collaboration, Canon Medical Systems, No.10 Huaxia Road, Guangzhou, Guangdong 510623 China

**Keywords:** Endovascular aortic repair, CT angiography, Deep learning, Artifacts, Iterative reconstruction

## Abstract

**Background:**

The image quality of computed tomography angiography (CTA) images following endovascular aneurysm repair (EVAR) is not satisfactory, since artifacts resulting from metallic implants obstruct the clear depiction of stent and isolation lumens, and also adjacent soft tissues. However, current techniques to reduce these artifacts still need further advancements due to higher radiation doses, longer processing times and so on. Thus, the aim of this study is to assess the impact of utilizing Single-Energy Metal Artifact Reduction (SEMAR) alongside a novel deep learning image reconstruction technique, known as the Advanced Intelligent Clear-IQ Engine (AiCE), on image quality of CTA follow-ups conducted after EVAR.

**Materials:**

This retrospective study included 47 patients (mean age ± standard deviation: 68.6 ± 7.8 years; 37 males) who underwent CTA examinations following EVAR. Images were reconstructed using four different methods: hybrid iterative reconstruction (HIR), AiCE, the combination of HIR and SEMAR (HIR + SEMAR), and the combination of AiCE and SEMAR (AiCE + SEMAR). Two radiologists, blinded to the reconstruction techniques, independently evaluated the images. Quantitative assessments included measurements of image noise, signal-to-noise ratio (SNR), contrast-to-noise ratio (CNR), the longest length of artifacts (AL), and artifact index (AI). These parameters were subsequently compared across different reconstruction methods.

**Results:**

The subjective results indicated that AiCE + SEMAR performed the best in terms of image quality. The mean image noise intensity was significantly lower in the AiCE + SEMAR group (25.35 ± 6.51 HU) than in the HIR (47.77 ± 8.76 HU), AiCE (42.93 ± 10.61 HU), and HIR + SEMAR (30.34 ± 4.87 HU) groups (*p* < 0.001). Additionally, AiCE + SEMAR exhibited the highest SNRs and CNRs, as well as the lowest AIs and ALs. Importantly, endoleaks and thrombi were most clearly visualized using AiCE + SEMAR.

**Conclusions:**

In comparison to other reconstruction methods, the combination of AiCE + SEMAR demonstrates superior image quality, thereby enhancing the detection capabilities and diagnostic confidence of potential complications such as early minor endleaks and thrombi following EVAR. This improvement in image quality could lead to more accurate diagnoses and better patient outcomes.

**Supplementary Information:**

The online version contains supplementary material available at 10.1186/s12880-024-01343-z.

## Introduction

Endovascular aneurysm repair (EVAR) is a minimally invasive treatment for abdominal aortic aneurysms [1]. Post-EVAR computed tomography angiography (CTA) monitoring is crucial for assessing stent patency and potential complications such as endoleaks [2], but metal artifacts from implants can significantly degrade image quality [3, 4]. Therefore, reducing metal artifacts has become important for enhancing diagnostic confidence and reducing the risk of missing underlying complications.

Metal Artifact Reduction (MAR) techniques such as Dual-Energy CT (DECT) and algorithms such as Single-Energy Metal Artifact Reduction (SEMAR) are available for this purpose [5–10]. DECT, a two-energy level scanning technique, effectively reduces metal artifacts with virtual mono-energetic images, but its clinical application is limited by complex workflows, time-consuming post-processing, and relatively high costs [6, 11, 12]. Studies also showed that there were some adverse factors for DECT in CTA follow-ups of EVAR due to low subjective diagnostic value and impaired endoleak visualization [11, 12]. In addition, the study of Mocanu et al. showed that DECTA was not helpful in intracranial aneurysms treated by coiling, a commonly used therapeutic method in the treatment of aneurysms [13]. Using clinical scanning data and tailored algorithms, SEMAR mitigates metal artifacts [14–16]. What’s more, Pan et al. showed that SEMAR reconstruction can improve CT image quality and may ultimately improve the detection of postoperative complications and patient prognosis [17]. But Ragusi et al. showed that it enhances CTA image quality in complex EVAR patients, but the metal density increases in some areas, affecting stent visualization [5]. Thus, SEMAR seems insufficient for post-EVAR CTA examinations and further enhancement is needed. In summary, there is still room in improvement for MAR techniques using in the EVAR postoperative CTA evaluation in terms of aspects such as process simplification, time reduction, image subjective evaluation, endoleak and stent visualization, and the removal of artifacts caused by coils.

In addition to artifacts, image noise is also a topic of clinically considerable concern in the evaluation of CTA follow-up exams. A high level of image noise can bring about adverse effects, such as low-level image quality, image segmentation difficulties [18], obscuring important details, making diagnosis more difficult, and affecting clinical decision. Currently, there are numerous methods that can mitigate the inconveniences caused by image noise. For example, the method, known as stochastic resonance theory, constructively uses the noise to enhance the signal, but this technique is mostly studied in optimizing the image registration and segmentation, not in image reconstruction [18–20]. Additionally, Deep Learning Reconstruction (DLR) has gained attention in the radiology field for reducing noise and enhancing image quality [21]. The Advanced Intelligent Clear-IQ Engine (AiCE, Canon Medical Systems) is a DLR application trained on iterative algorithms with a deep learning neural network, which is already commercialized but currently only available for Canon machines. Previous studies showed it embraces many outstanding advantages such as enhancing density and spatial resolution, reducing artifacts and noise, and creating extraordinarily high-quality images without increasing radiation dose [22–24]. In a phantom study, AiCE plus SEMAR performed the best in reducing artifacts compared to other algorithms [25]. Moreover, studies have shown that combining DLR with SEMAR further reduces image noise and artifacts, thus improving image quality, in CT images with metal implants [26, 27]. However, the usefulness of AiCE with SEMAR (AiCE + SEMAR) in EVAR patients has not been reported. Therefore, we hypothesize that the combination of these two technologies will play a surprisingly significant role in reducing artifacts and improving image quality during CTA evaluation after EVAR surgery, likely to solve the dilemma we mentioned above.

To verify our hypothesis, this study would assess the usefulness of the AiCE + SEMAR algorithm in EVAR postoperative CTA evaluation by comparing it with that of standalone AiCE, hybrid iterative reconstruction (HIR, the standard clinical protocol at our institution), and the combination of HIR plus SEMAR (HIR + SEMAR) from both qualitative and quantitative aspects. In terms of qualitative aspect, several aspects including the overall image quality, the stent and coil artifacts, and the visualization of stent and adjacent structures would be evaluated. In terms of quantitative aspect, several quantitative indicators to evaluate metal artifacts and image quality, such as CT attenuation value (CT), standard deviation (SD), contrast-to-noise ratio (CNR), signal-to-noise ratio (SNR), artifact index (AI) and the longest length of artifacts (AL), would be analyzed. Extra above quantitative analysis for patients with coil embolization would also be carried out. In addition, the value of AiCE + SEMAR algorithm in detecting postoperative complications of EVAR, such as endoleak and in-stent thrombosis, would been further discussed through several clinical CTA cases. The flowchart displaying research process is shown in Supplemental Fig. 1.

## Methods

### Patients

This retrospective, single-center study was approved by our Research Ethics Committee, and the requirement for formal informed consent was waived. All patients provided consent for contrast-enhanced CT scans. This study focused on patients who underwent CTA examinations of the abdominal aorta and bilateral iliac arteries at our hospital from March to June 2023. The inclusion criteria were as follows: (1) patients who were scanned on a certain machine with SEMAR and AiCE algorithms; (2) patients who had already undergone abdominal aortic aneurysm repair surgery; and (3) patients who received Y-shaped stent implantation. The exclusion criteria were as follows: (1) patients with severe cardiac, renal failure and severe iodine allergy; (2) patients with stent graft infections; (3) images with insufficient data or significant motion artifacts on any scan. Supplemental Fig. 2 depicts the inclusion-exclusion procedure, and Table 1 describes the characteristics of the enrolled patients. Some of the finally enrolled patients were with internal iliac artery coil embolization, endoleak or in-stent thrombosis, on which we conducted additional analysis. Notably, three male patients, aged 70, 73, and 77 years, underwent repeated CTA tests due to embolization within the stent or endoleak. It is worth mentioning that subsequent statistical analysis treated these repeated tests as independent instances.

**Table 1:** Patient characteristics

Characteristics	Values
No. of patients	44
CTA scans	47
Age (y), mean ± SD	68.6 ± 7.8
Sex (male/female)	37/7
BMI (Kg/m^2^), mean ± SD	23.3 ± 2.8
Category one: Coils for internal iliac artery embolization	
No. of patients on the right side	12
No. of patients on the left side	7
No. of patients on bilateral sides	3*
total CTA scans	25
Category two: Endoleaks	
No. of patients with type I	8
No. of patients with type II	3
No. of patients with type III	2
total CTA scans	13
Category three: In-stent thrombosis	
total CTA scans	4

*Abbreviation****BMI*** mean body mass index

*Note* *These have been counted as 6 independent CTA scans. And there were also some scans that didn’t belong to above three categories and were not separately listed

### CT scanning and reconstruction

All patients were scanned using a 320-row, 640-slice multidetector CT scanner (Aquilion ONE GENESIS; Canon Medical Systems, Japan). The detailed scanning protocols are provided in Supplemental Appendix 1. After scanning, four volumetric CT reconstructions were performed for each patient according to the following protocols: (1) HIR algorithm; (2) AiCE algorithm; (3) HIR plus SEMAR algorithm; and (4) AiCE plus SEMAR algorithm.

### Qualitative image analyses

Two expert radiologists (with 10 and 13 years of experience in CT) blindly and independently evaluated the CT images using identical window levels and width settings for each scan. One of the radiologists reviewed the images twice. However, in order to prevent recollection bias, a gap of at least one month existed between the reviews. Subjective visual scores (ranging from 1 [worst] to 5 [best]) were used to assess various aspects of image quality (detailed in Table 2). Artifact severity was also assessed using a 5-point scale for patients who underwent embolization of the internal iliac arteries with coils (Table 2).

**Table 2:** The 5-point scale criteria for subjective evaluation

Scores	overall image quality	visibility of surrounding organs	in-stent vessels on the most severe artifact scan	out-stent thrombus on the most severe artifact scan	artifact on the most severe coils artifact scan
5	excellent image quality, free of artifacts, high diagnostic confidence	excellent image quality, free of artifact interference	basically free of artifacts, excellent visualization of in-stent vessels	basically free of artifacts, excellent visualization of out-stent thrombus	basically free of artifacts, excellent visualization of internal and external iliac arteries
4	good image quality, minor artifacts, fully evaluable and diagnostic	good image quality, free of artifact interference	minor artifacts, good visualization of in-stent vessels	minor artifacts, good visualization of out-stent thrombus	minor artifacts, good visualization of internal and external iliac arteries
3	adequate image quality, moderate artifacts, acceptable for diagnosis	acceptable image quality, slightly obscured (< 20%), minor artifacts, interference	many artifacts, adequate visualization of in-stent vessels	many artifacts, adequate visualization of out-stent thrombus	many artifacts, adequate visualization of internal and external iliac arteries
2	suboptimal image quality, many artifacts, partly for diagnosis	suboptimal image quality, partially obscured (20–50%), many artifacts interference	many artifacts, poor visualization of in-stent vessels	many artifacts, poor visualization of out-stent thrombus	many artifacts, poor visualization of internal and external iliac arteries
1	poor image quality, major artifacts, nondiagnostic confidence	poor image quality, severely obscured (> 50%), major artifacts interference	major artifacts, blurred visualization of in-stent vessels	major artifacts, blurred visualization of out-stent thrombus	major artifacts, blurred visualization of internal and external iliac arteries

### Quantitative image analyses

**Fig. 1:**
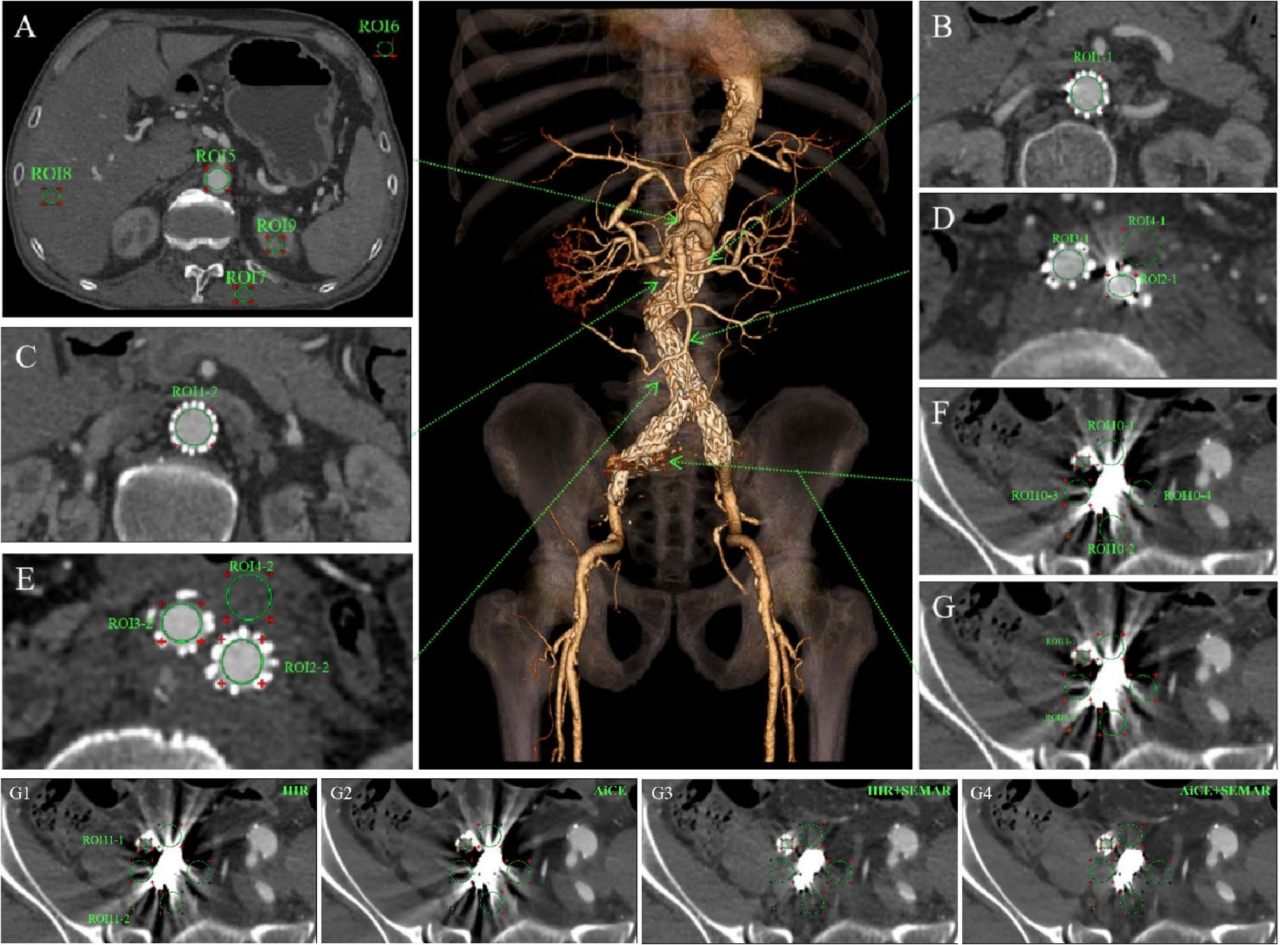
Schematic illustration of the ROI position. CTA was performed on a 70-year-old man who underwent endovascular repair of an abdominal aortic aneurysm and right internal iliac artery embolization utilizing a coil. (**A-G**) Corresponding axial views at the level indicated by arrows in the volume-rendered image (the middle image). The four reconstruction methods for G1-G4 were HIR, AiCE, HIR + SEMAR, and AiCE + SEMAR

Quantitative image analysis was conducted using RadiAnt DICOM Viewer software (Medixant, Poland, https://www.radiantviewer.com/, version 2021.2). Figure 1 depicts an example of several regions of interest (ROIs) placement and naming conventions. ROIs were placed on axial scans with the most severe or mild abnormalities, including the main body (ROI1-1, ROI1-2), left common iliac branched section (ROI2-1, ROI2-2), and right common iliac branched section (ROI3-1, ROI3-2) of the stent repairing the abdominal aortic aneurysm. Additional ROIs were placed on areas with thrombi outside the stent (ROI4-1, ROI4-2), with or without artifacts, and on various anatomical structures, such as the abdominal aorta (ROI5), air (ROI6), erector spinae muscle (ROI7), liver (ROI8), and kidney (ROI9). For patients who underwent internal iliac artery embolization using coils, ROIs were also set around the coil at different positions (ROI10-1 - ROI10-4). On the current layer, ALs were measured, and ROIs were placed adjacent to the internal and external iliac arteries (ROI11-1, ROI11-2). CT and SDs were recorded for each ROI. SNRs, CNRs, and AIs were calculated by applying the following formulas:

1. SNR = CT_target_/SD_reference_, where CT_target_ refers to the mean Hounsfield scale of the estimated tissue, and SD_reference_ is the standard deviation for Hounsfield scales of air.
2. $\text{C}\text{N}\text{R}=\frac{\left|{\text{C}\text{T}}_{\text{t}\text{a}\text{r}\text{g}\text{e}\text{t}}-{\text{C}\text{T}}_{\text{r}\text{e}\text{f}\text{e}\text{r}\text{e}\text{n}\text{c}\text{e}}\right|}{\sqrt{\left({\text{S}\text{D}}_{\text{t}\text{a}\text{r}\text{g}\text{e}\text{t}}^{2}-{\text{S}\text{D}}_{\text{r}\text{e}\text{f}\text{e}\text{r}\text{e}\text{n}\text{c}\text{e}}^{2}\right)/2}}$, in which CT_target_ and CT_reference_ refer to the Hounsfield scale of the estimated tissue and erector spinae muscle, respectively, and SD_target_ and SD_reference_ represent the mean and standard deviation of the Hounsfield scale of the estimated tissue and erector spinae muscle, respectively.
3. $\text{A}\text{I} =\sqrt{{\text{S}\text{D}}_{\text{a}\text{r}\text{t}\text{i}\text{f}\text{a}\text{c}\text{t}}^{2}-{\text{S}\text{D}}_{\text{r}\text{e}\text{f}\text{e}\text{r}\text{e}\text{n}\text{c}\text{e}}^{2}}$, in which SD_artifact_ and SD_reference_ referred to the mean and standard deviation of the Hounsfield scale of the estimated tissue with artifact and erector spinae muscle, respectively.

### Statistical analysis

The data were analyzed using the SPSSAU data scientific analysis platform (QingSi Technology Ltd, China, https://spssau.com) [23] and GraphPad Prism (GraphPad Prism 8; GraphPad Software Inc, USA). Descriptive data were presented as the mean ± SD. One-way analysis of variance (ANOVA) with post hoc Tukey’s or Dunn’s pairwise comparisons were performed to analyze qualitative and quantitative data, respectively. Statistical significance was set at *p* < 0.05 or *p* < 0.008 (adjusted for multiple comparisons). Intra- and interobserver reliability were assessed using intraclass correlation coefficients (ICCs) with two-way random, single measures of absolute agreement. The corresponding 95% confidence intervals (CIs) and p values are reported.

## Results

### Qualitative evaluation

**Fig. 2:**
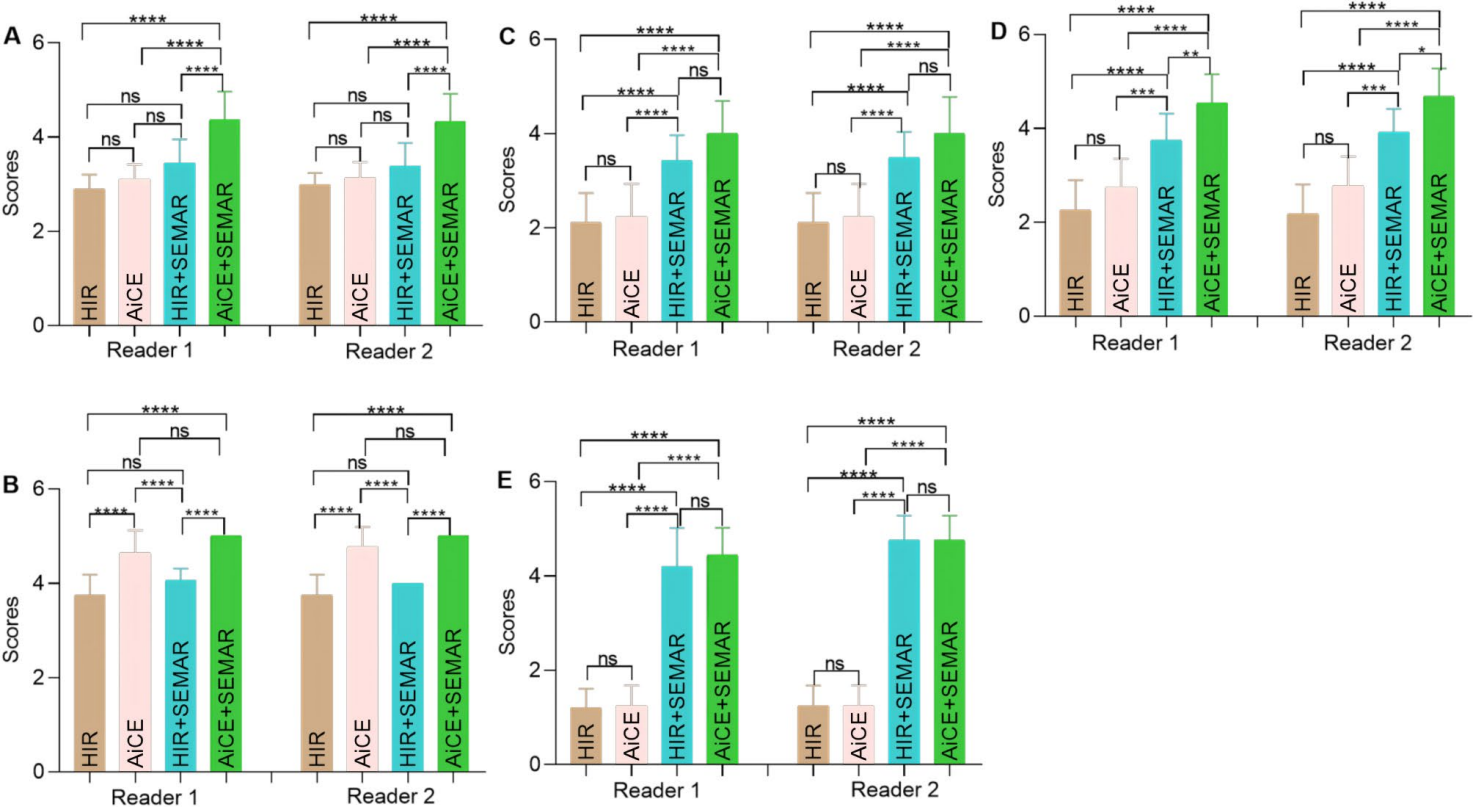
Detailed subjective image analysis results. (**A**) Overall image quality; (**B**) Visibility of the surrounding organs; In-stent vessels (**C**) and out-stent thrombus (**D**) on the most severe artifact scan; (**E**) Artifacts on the most severe coil artifact scan. Note. ns: adjusted *p* > 0.05; *: adjusted *p* < 0.05; **: adjusted *p* < 0.01; ***: adjusted *p* < 0.001; ***: adjusted *p* < 0.0001. The Dunn method was used for post hoc pairwise comparisons

**Fig. 3:**
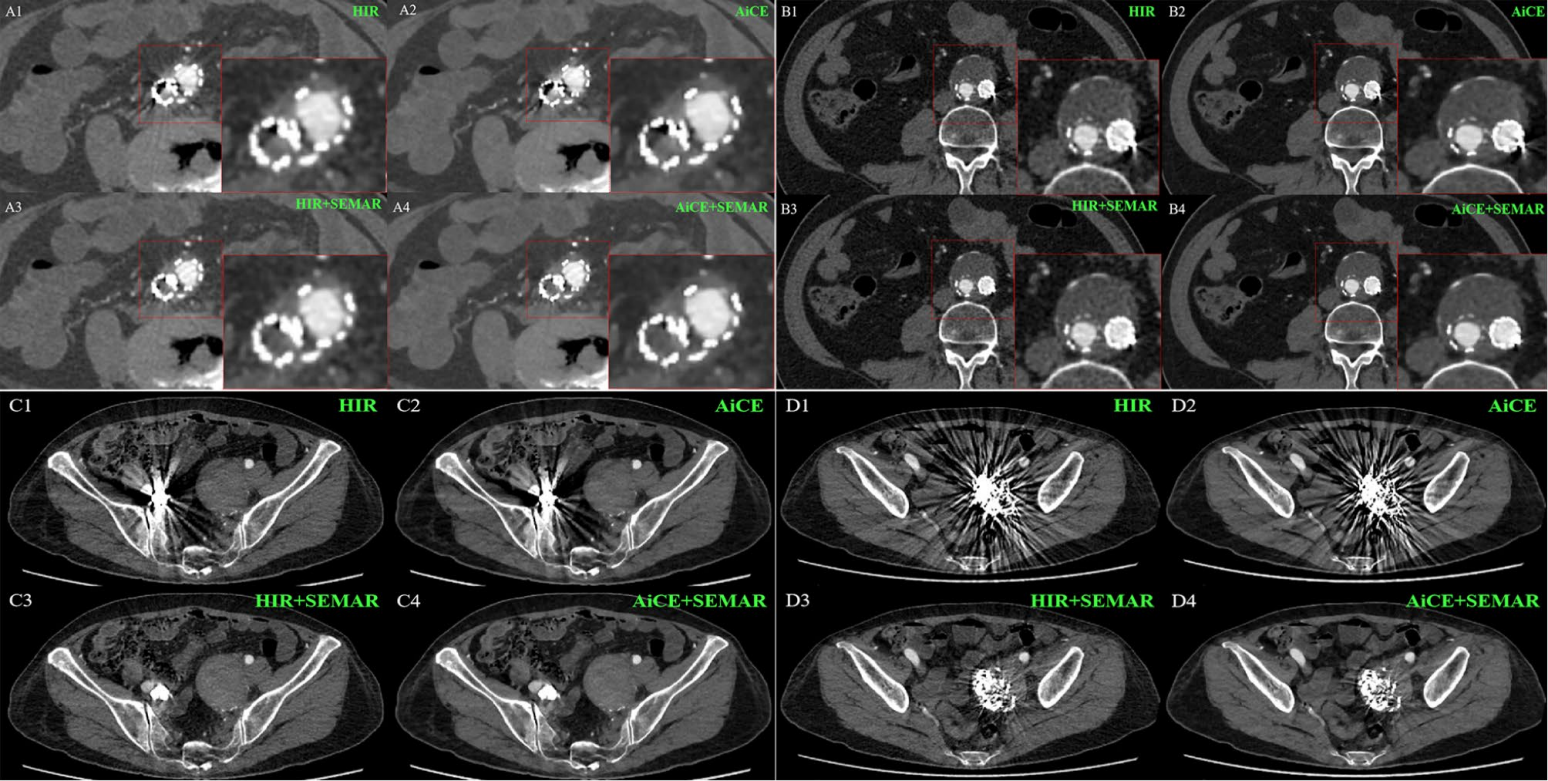
Exemplary axial CTA images with different reconstructions for qualitative evaluation. (A1-A4) A 73-year-old woman. Reader 1 and 2 scored the degree of in-stent vessels on the most severe artifact scan as 1/1/3/3. (B1-B4) A 66-year-old man. The degree of in-stent vessels on the most severe artifact scan was rated as 1/1/3/3 by readers 1 and 2, and the degree of out-stent thrombus on the most severe artifact scan was rated as 2/2/4/5 by readers 1 and 2. (C1-C4) A 70-year-old woman. Both Reader 1 and Reader 2 scored the degree of artifacts on the most severe coil artifact scan as 1/1/5/5. (D1-D4) A 70-year-old woman. The degree of artifacts on the most severe coil artifact scan was rated as 1/1/3/4 by Reader 1 or 1/1/4/4 by Reader 2. The block in the lower left corner of a single image (A1-A4, B1-B4) represents the local amplification of the respective places

The subjective image analysis results are summarized in Table 3; Fig. 2. Both the interobserver agreement between the two readers and the intraobserver agreement of Reader 2 were excellent (ICC > 0.9) across all aspects of image quality evaluation. Consistently, both readers observed significant variations in image quality among the four reconstruction methods (*p* < 0.0001). Compared with the remaining three groups, the AiCE + SEMAR group demonstrated superior overall image quality (*p* < 0.001). Moreover, compared with the absence of AiCE, the application of the AiCE algorithm significantly improved the visibility of adjacent organs (*p* < 0.001). However, the SEMAR algorithm did not enhance the visibility of neighboring organs (*p* > 0.05). On the scan exhibiting the most pronounced metal artifacts, HIR + SEMAR and AiCE + SEMAR significantly reduced these artifacts (*p* < 0.0001) and facilitated better visualization of the in-stent vessels and out-stent thrombus (*p* < 0.0001 or *p* < 0.001). Nevertheless, no discernible differences were observed between HIR and AiCE or between HIR + SEMAR and AiCE + SEMAR, except for thrombus formation outside the stent (*p* < 0.05). Representative images, incorporating subjective evaluation scores, are presented in Fig. 3, highlighting the superior image quality achieved with AiCE + SEMAR, characterized by minimized imaging artifacts and enhanced visualization of surrounding soft tissues. Specifically, Fig. 3C4 and 3D4 exhibited marked improvement in the visibility of soft tissue around the graft, including the adjacent ileum, muscles, sacrum, and abdominal wall, compared to other reconstruction methods.

**Table 3:** Detailed results of subjective image analysis

	Reader 1				Reader 2				^ANOVA comparisons (^*p* ^− value)^		^Intraobserver agreement^(^Reader 2)^	Interobserver agreement
	HIR	AiCE	HIR + SEMAR	AiCE + SEMAR	HIR	AiCE	HIR + SEMAR	AiCE + SEMAR				
quantitative content	(1/2/3/4/5) [total scores]				(1/2/3/4/5) [total scores]				Reader 1	Reader 2	ICC (95%CI)	ICC (95%CI)
overall image quality (*n* = 47)	0/5/42/0/0 [136]	0/0/42/5/0 [146]	0/0/26/21/0 [162]	0/03/24/20 [205]	0/2/44/1/0 [140]	0/0/41/6/0 [147]	0/0/29/18/0 [159]	0/0/3/26/ 18 [203]	< 0.0001****	< 0.0001****	0.983 (0.977 ~ 0.987)	0.935 (0.914 ~ 0.951)
visibility of surrounding organs (*n* = 47)	0/0/12/35/0 [176]	0/0/0/17/30 [218]	0/0/0/44/3 [191]	0/0/0/0/47 [235]	0/0/12/35/0 [176]	0/0/0/11/36 [224]	0/0/0/47/0 [188]	0/0/0/0/ 47 [235]	< 0.0001****	< 0.0001****	0.983 (0.977 ~ 0.988)	0.935 (0.914 ~ 0.952)
in-stent vessels on the most severe artifact scan (*n* = 47)	5/34/6/2/0 [99]	5/28/12/2/0 [105]	0/0/28/18/1 [161]	0/0/11/25/11 [188]	5/34/6/2/0 [99]	5/28/12/2/0 [105]	0/0/25/21/1 [164]	0/0/14/19/ 14 [188]	< 0.0001****	< 0.0001****	0.983 (0.977 ~ 0.989)	0.935 (0.914 ~ 0.953)
out-stent thrombus on the most severe artifact scan (*n* = 47)	4/28/14/1/0 [106]	1/13/30/3/0 [129]	0/0/15/29/3 [176]	0/0/3/16/28 [213]	5/30/11/1/0 [102]	2/10/32/3/0 [130]	0/0/8/35/4 [184]	0/0/3/9/ 35 [220]	< 0.0001****	< 0.0001****	0.983 (0.977 ~ 0.990)	0.935 (0.914 ~ 0.954)
artifacts on the most severe coils artifact scan (*n* = 25)	20/5/0/0/0 [30]	19/6/0/0/0 [31]	0/0/6/8/11 [105]	0/0/1/12/12 [111]	19/6/0/0/0 [31]	19/6/0/0/0 [31]	0/0/1/4/20 [119]	0/0/1/4/ 20 [119]	< 0.0001****	< 0.0001****	0.983 (0.977 ~ 0.991)	0.935 (0.914 ~ 0.955)

*Abbreviation****ANOVA*** One-way analysis of variance, ***ICC*** intra-class correlation coefficients, ***CI*** confidence intervals

### Quantitative evaluation

**Fig. 4:**
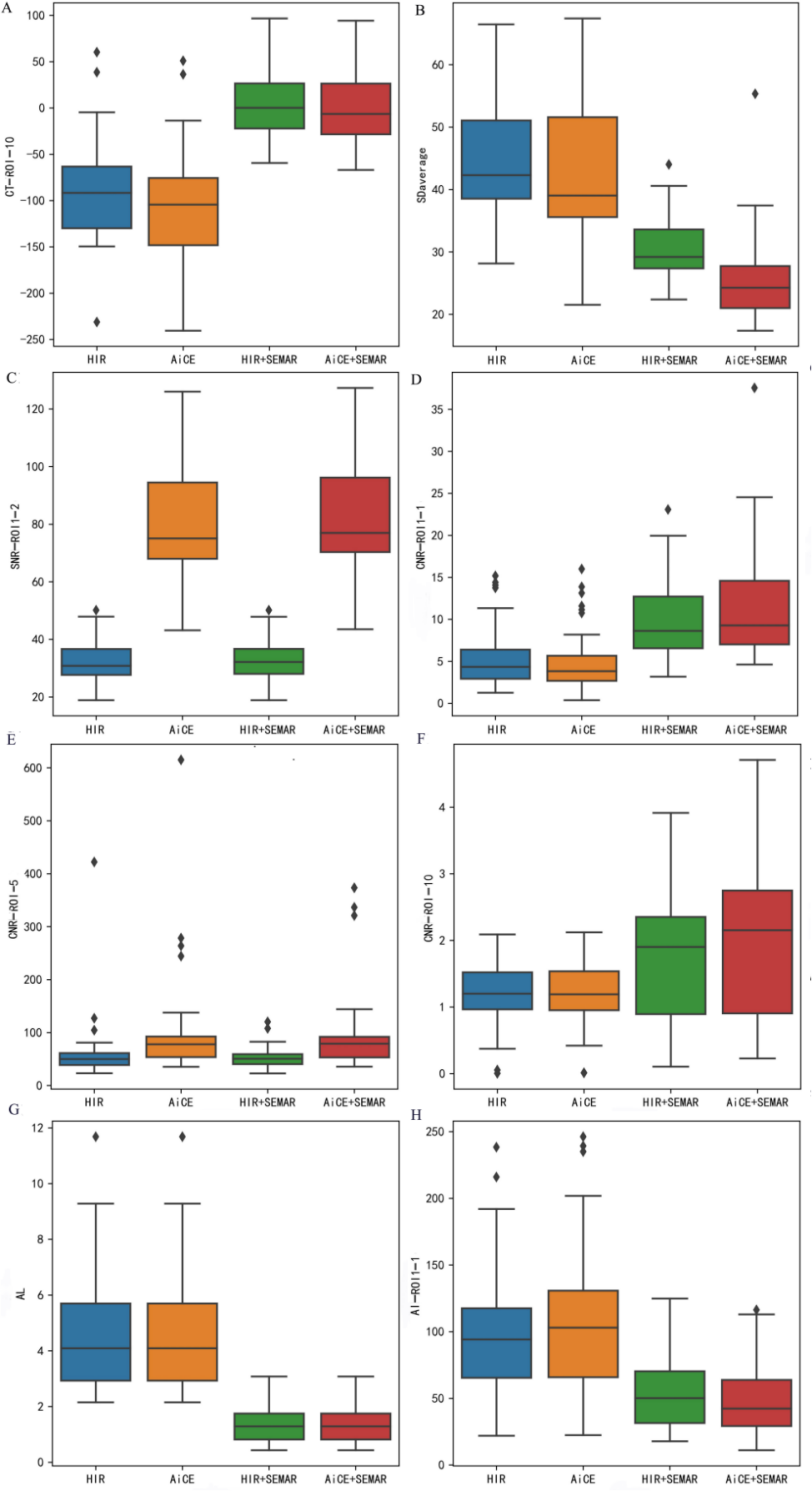
Representative results of quantitative analysis depicted as box plots for different indicators. (**A**) CT image of ROI10; (**B**) SD_average_; (**C**) SNR of ROI1-2; (**D**) CNR of ROI1-1; (**E**) CNR of ROI5; (**F**) CNR of ROI10; (**G**) AL values; (**H**) AI of ROI1-1. The unit of CT, and SD values is HU, and the unit of AL values is cm

**Fig. 5:**
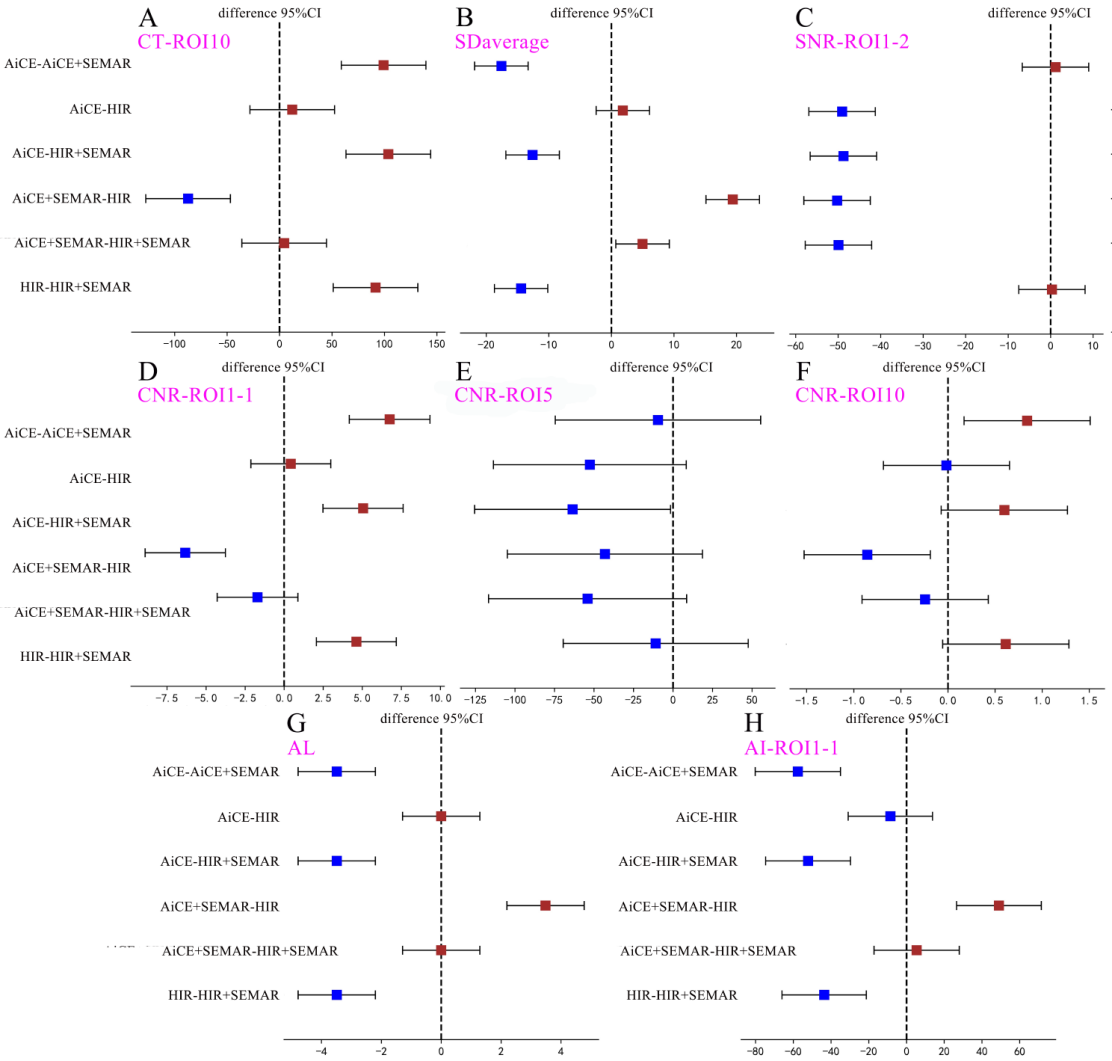
Results of post hoc pairwise comparisons among quantitative analysis of the four reconstructions depicted as forest plots for different indicators. (**A**) CT values of ROI10; (**B**) SD_average_; (**C**) SNR of ROI1-2; (**D**) CNR of ROI1-1; (**E**) CNR of ROI5; (**F**) CNR of ROI10; (**G**) AL values; (**H**) AI of ROI1-1. The unit of CT, and SD values is HU, and the unit of AL values is cm

The results of objective image analysis using HIR, AiCE, HIR + SEMAR, and AiCE + SEMAR are displayed in Figs. 4 and 5, Supplemental Fig. 3, Supplemental Fig. 4, Supplemental Tables 1, and Supplemental Table 2.

### CT attenuation values

The HIR + SEMAR and AiCE + SEMAR groups exhibited greater CT attenuation values than did the HIR and AiCE groups (*p* < 0.008, Figs. 4A and 5A). Nevertheless, no significant differences were noticed in CT attenuation values when comparing HIR + SEMAR with AiCE + SEMAR between ROI1-1 and ROI1-2 or between ROI4-1 and ROI4-2 (*p* > 0.05).

### Image noise

The mean noise levels, calculated as the average SD derived from ROIs ranging from ROI1-1 to ROI9, were 47.77 ± 8.76, 42.93 ± 10.61, 30.34 ± 4.87, and 25.35 ± 6.51 HU for CT images reconstructed using HIR, AiCE, HIR + SEMAR, and AiCE + SEMAR, respectively (Fig. 4B, *p* < 0.001). Compared to those of the HIR, the noise of the AiCE, HIR + SEMAR, and AiCE + SEMAR models decreased by 10.13%, 36.49%, and 46.93%, respectively. A significant reduction in noise was observed when SEMAR was applied, particularly when SEMAR was combined with AiCE (all *p* < 0.008, Fig. 5B). These findings were consistent in patients who received coils for embolization of the internal iliac artery (all *p* < 0.008).

### Signal-to-noise ratio (SNR)

The majority of ROIs demonstrated significantly greater SNRs with the application of AiCE than with the application of HIR or HIR + SEMAR (all *p* < 0.008, Figs. 4C and 5C). However, SEMAR did not contribute to enhancing the SNR (*p* > 0.05, Fig. 5C). Notably, AiCE + SEMAR achieved the highest SNR (Fig. 4C). Only ROI1-1, ROI4-1, and ROI10 showed statistically significant improvements in the SNR with the application of SEMAR.

### Contrast-to-noise ratio (CNR)

The values of CNRs significantly differed among the four reconstruction techniques (*p* < 0.001) in the following descending order: AiCE + SEMAR, HIR + SEMAR, AiCE, and HIR (Fig. 4D and E, and 4F). However, the pairwise comparisons revealed no significant differences between any two groups for ROI5 (*p* > 0.05, Fig. 5E) or between AiCE and HIR or between AiCE + SEMAR and HIR + SEMAR for ROI1-1, ROI2-1, and ROI3-1 (*p* > 0.05, Fig. 5D). The only significant pairwise difference in ROI10 was observed between the AiCE + SEMAR group (2.01 ± 1.24) and the HIR group (1.15 ± 0.55) (*p* < 0.008, Figs. 4F and 5F).

### Longest length of the artifacts (AL)

The ALs in the CT images reconstructed with the HIR, AiCE, HIR + SEMAR, and AiCE + SEMAR methods were 4.81 ± 2.39, 4.81 ± 2.39, 1.33 ± 0.61, and 1.33 ± 0.61 cm, respectively, indicating that the application of the SEMAR significantly reduced the AL (*p* < 0.05, Figs. 4G and 5G).

### Artifact index (AI)

The results revealed that the administration of SEMAR significantly decreased the AIs (*p* < 0.008, Figs. 4H and 5H), with a further reduction when AiCE and SEMAR were applied together (*p* < 0.008, Figs. 4H and 5H). Notably, there were no significant differences in the AIs between AiCE and HIR or between AiCE + SEMAR and HIR + SEMAR (*p* > 0.05, Fig. 5H).

### Lesion detection

As evident in Fig. 3A1, using routine scanning, dark streaks from the stent obscured the depiction of adjacent tissues, making any anomalies uncertain. However, the combined application of AiCE and SEMAR reduced the number of dark bands and enabled a clear depiction of stent thrombosis within the stent, as shown in Fig. 3A4, highlighting the efficacy of AiCE + SEMAR in identifying in-stent thrombosis and evaluating stent patency.

**Fig. 6:**
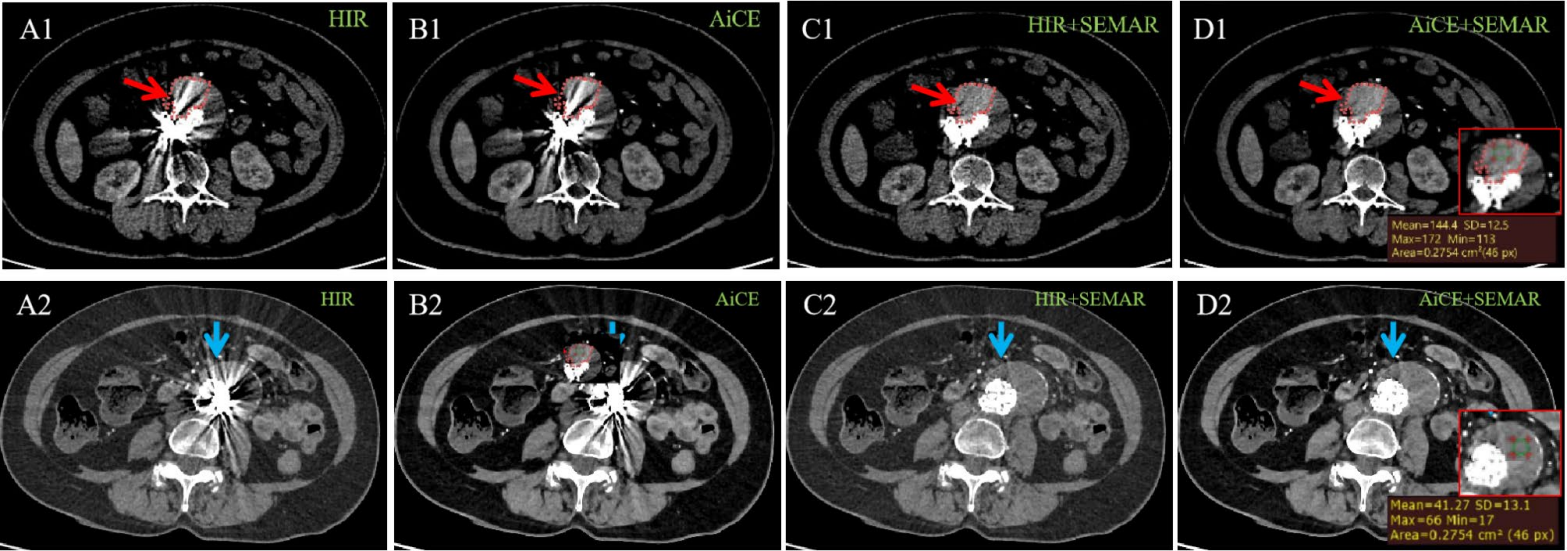
Excellent visualization of the endoleak with AiCE + SEMAR reconstruction. Axial CTA images were obtained using the HIR (A1, A2), AiCE (B1, B2), HIR + SEMAR (C1, C2), and AiCE + SEMAR (D1, D2) techniques. The images on the top row (A1-D1) represent the case of the endoleak, while the images on the bottom row (A2-D2) represent the case without the endoleak. The arrows represent the false lumen outside stents, and the red circles in the abdominal aorta (A1-D1) indicate the extent of contract leakage. The images within the red box at the bottom left corner (D1, D2), in which ROIs are put to display CT values, are the local enlarged views of the lesion areas

Moreover, as depicted in Fig. 6 and Supplemental Fig. 5, the identification of endoleaks in images reconstructed with HIR alone posed a challenge due to the bright streaks from implants. Encouragingly, the implementation of AiCE + SEMAR (as shown in Fig. 6D1) further minimized the bright bands, resulting in a clear visualization of the contrast agent that leaked out and a distinct delineation between the thrombus and contrast agent. In comparison with images for false lumen without endoleak shown as Fig. 6D2, images with endoleak using AiCE + SEMAR (Fig. 6D1) visually exhibited a slightly more density for false lumen and a density contrast between thrombosis and leaked contrast agent, rendering it more visually discernible and thereby facilitating earlier detection of endoleak. This improved visual clarity was verified by the improved CT value of the ROI in Fig. 6D1 indeed, and the endoleak was subsequently corroborated by DSA examination (Supplemental Video 1), leading to increased diagnostic confidence and accurate assessment of endoleak severity.

## Discussion

This study tested the hypothesis that the combination of SEMAR and the novel DLR algorithm, AiCE, can complementarily and effectively improve the quality of CTA images during EVAR follow-up. As expected, our findings revealed that compared to other reconstruction techniques, the AiCE + SEMAR algorithm significantly reduced artifacts (nearly three-quarters reduction) and image noise (maximum nearly half reduction) while enhancing the CNR and SNR, indicating improved contrast resolution, structural visibility and image quality. Notably, this approach significantly improved the early detection of endoleaks and thrombi, which provides clinicians with an optimized valuable tool for quantitative evaluations of internal leakage, thereby enabling them to formulate optimal treatment protocols, such as intervening slightly earlier and more effectively with improved survival.

Clear visualization, both within and beyond the stent, was crucial for detecting leakage and thrombosis after EVAR, while bright and dark bands from grafts prevented the oversight. Kazimierczak et al. showed that there was a decrease in the parameters of qualitative image assessment and low subjective diagnostic value for CTA follow-ups of EVAR using DECT [12]. But delightfully, our subjective evaluation demonstrated that the combination of AiCE and SEMAR effectively mitigated metal artifacts to the greatest extent in cases with not only stent grafts but also coils embolization, leading to improved overall image quality. This enhancement facilitated better image interpretability and diagnostic certainty. Furthermore, the strong inter- and intraobserver consistency observed highlighted the reliability and practicality of our subjective evaluation. Hence, this technology exhibits promising clinical utility for assisting radiologists in the early detection of mild lesions obscured by metal artifacts, thereby bolstering diagnostic certainty, obviating the need for invasive confirmatory procedures, facilitating prompt and early intervention, and enabling the formulation of tailored treatment strategies, thereby potentially improving patient survival rates.

To objectively assess the performance of AiCE + SEMAR in detecting potential stent-related lesions, we selected numerous ROIs from various graft levels. Analysis indicated that CT attenuation was primarily affected at specific levels where artifacts were prominent. However, the application of SEMAR significantly diminished or even negated this effect, consistent with previous reports [14, 28]. Consequently, this approach proved suitable for standard criteria and morphological evaluations based on CT attenuation.

Although noise levels progressively decreased in the order of HIR, AiCE, HIR + SEMAR, and AiCE + SEMAR sequences, no statistically significant difference was observed between HIR and AiCE. This finding contradicts previous literature [29]. We hypothesized that artifacts interfered with the potential noise reduction capabilities of AiCE despite the artifact mitigation ability of SEMAR. Therefore, we explored noise levels in regions with minimal artifacts, specifically ROI1-2 and ROI2-2. Our results showed that noise levels were lower with AiCE than that without it, and interestingly, AiCE produced notably less noise than HIR + SEMAR. There was no statistically significant difference between AiCE and AiCE + SEMAR or between HIR and HIR + SEMAR, suggesting that the noise reduction capabilities of SEMAR may be limited in the absence of artifacts and that AiCE was primarily responsible for noise reduction.

In terms of the SNR and CNR, at the level severely affected by artifacts, which was our primary concern, the combination of AiCE and SEMAR yielded the best results. This suggests superior image quality and enhances the detection of diverse lesions, particularly those with low contrast [30]. This observation was further validated in patients with early endoleaks and thrombus within the stent. While AiCE + SEMAR significantly reduced artifacts in the assessments of AL and AI comparing to others, AiCE alone had a minimal artifact reduction effect.

Overall, this study revealed that AiCE and SEMAR played eminent and complementary roles in varying degrees and levels of metal artifacts for different regions of the target images. At the level with numerous artifacts, SEMAR was primarily responsible for both artifact and noise reduction, whereas AiCE primarily increased the effect of noise reduction. At the level with minor or no artifacts, only AiCE was primarily responsible for noise reduction. Therefore, the powerful combination of the two methods can contribute to a more comprehensive and better evaluation of the images of the same patient. In detail, the AiCE plus SEMAR reconstruction algorithm overall performed the best in multiple aspects of image assessment, including excellent image quality, significant artifact and noise reduction, enhanced visualization of stent patency, and optimized evaluation of surrounding structures, such as endoleaks and thrombosis.

Practically speaking, the reconstruction time for AiCE + SEMAR was remarkably short in comparison with that for DECT [6], less than 2 min, which is comparable to the time required for radiographers to move patients from the examination bed. This suggests that the AiCE + SEMAR technique is feasible and could be integrated into routine clinical practice. Furthermore, previous studies have shown that both AiCE and SEMAR substantially reduce patient radiation exposure without compromising image quality [24, 31, 32], which was just the shortcomings of the methodology using DECT. The ability of AiCE + SEMAR to effectively minimize radiation exposure while maintaining image quality is promising and warrants further investigation. Nonetheless, our study has limitations, including its small sample size and single-center design, which may lead to potential bias such as sample bias. Patients who underwent EVAR at external medical facilities may present with varying medical conditions, surgical approaches, and implant types, potentially influencing the robustness of the findings in this study, though any disparities in these factors would probably have minimal impact on the overall results. Future research will be performed in multiple centers using a prospective design, in order to strengthen the conclusions we drawn and assess their broader generalizability and applicability.

## Conclusions

Compared to HIR, SEMAR, and HIR + SEMAR, the combination of AiCE and SEMAR demonstrated superior diagnostic performance and image quality in CTA evaluations following EVAR. This advantage facilitates the detection of potential complications, such as early endoleaks and thrombosis, enabling clinicians to develop optimal prompt treatment strategies. The complementary roles of the two methods contribute to a comprehensive evaluation of patient outcomes. Our study contributes to effective clinical practice and highlights the potential for further optimization in routine clinical settings. Future research will explore the use of AiCE and SEMAR in low-dose scans while maintaining image quality. Further analysis will be explored to better highlight the improved thrombus and endoleak detection with the optimized algorithms.

### Electronic supplementary material

Below is the link to the electronic supplementary material.

Supplementary Material 1

Supplementary Material 2

Supplementary Material 3

Supplementary Material 4

Supplementary Material 5

Supplementary Material 6

Supplementary Material 7

## Data Availability

The statistical data used to support the findings of this study are available from the corresponding author upon request.

## Abbreviations

CTA: CT angiography
HIR: hybrid iterative reconstruction
DLR: Deep Learning Reconstruction
AiCE: Advanced Intelligent Clear-IQ Engine
SEMAR: Single-Energy Metal Artifact Reduction
HIR + SEMAR: Combination of hybrid iterative reconstruction and Single-Energy Metal Artifact Reduction
AiCE + SEMAR: Combination of Advanced Intelligent Clear-IQ Engine algorithm and Single-Energy Metal Artifact Reduction
EVAR: Endovascular aortic repair
CNR: Contrast-to-noise ratio
SNR: Signal-to-noise ratio
AL: The longest length of artifacts
AI: Artifact index
ROI: Regions of interest

## References

[1] Secretariat MA (2002). Endovascular repair of abdominal aortic aneurysm: an evidence-based analysis. Ont Health Technol Assess Ser.

[2] Smith T, Quencer KB (2020). Best practice guidelines: imaging surveillance after endovascular aneurysm repair. AM J ROENTGENOL.

[3] Moshfeghi M, Safi Y, Różyło-Kalinowska I, Gandomi S (2022). Does the size of an object containing dental implant affect the expression of artifacts in cone beam computed tomography imaging?. Head Face Med.

[4] Sonnow L, Könneker S, Vogt PM, Wacker F, von Falck C (2017). Biodegradable magnesium Herbert screw - image quality and artifacts with radiography, CT and MRI. BMC Med Imagin.

[5] Ragusi M, van der Meer RW, Joemai R, van Schaik J, van Rijswijk C (2018). Evaluation of ct angiography image quality acquired with single-energy metal artifact reduction (semar) algorithm in patients after complex endovascular aortic repair. CARDIOVASC INTER RAD.

[6] Kazimierczak W, Kazimierczak N, Serafin Z. Review of clinical applications of dual-energy ct in patients after endovascular aortic repair. J CLIN MED 2023;12. 10.3390/jcm12247766 PMC10743598 38137834

[7] Katsura M, Sato J, Akahane M, Kunimatsu A, Abe O (2018). Current and novel techniques for metal artifact reduction at ct: practical guide for radiologists. Radiographics.

[8] Bolstad K, Flatabø S, Aadnevik D, Dalehaug I, Vetti N (2018). Metal artifact reduction in ct, a phantom study: subjective and objective evaluation of four commercial metal artifact reduction algorithms when used on three different orthopedic metal implants. ACTA RADIOL.

[9] Chou R, Chi HY, Lin YH, Ying LK, Chao YJ, Lin CH (2020). Comparison of quantitative measurements of four manufacturer’s metal artifact reduction techniques for ct imaging with a self-made acrylic phantom. TECHNOL HEALTH CARE.

[10] Greffier J, Larbi A, Frandon J, Daviau PA, Beregi JP, Pereira F (2019). Influence of iterative reconstruction and dose levels on metallic artifact reduction: a phantom study within four ct systems. DIAGN INTERV IMAG.

[11] Boos J, Fang J, Heidinger BH, Raptopoulos V, Brook OR (2017). Dual energy ct angiography: pros and cons of dual-energy metal artifact reduction algorithm in patients after endovascular aortic repair. ABDOM RADIOL.

[12] Kazimierczak W, Nowak E, Kazimierczak N, Jankowski T, Jankowska A, Serafin Z (2023). The value of metal artifact reduction and iterative algorithms in dual energy ct angiography in patients after complex endovascular aortic aneurysm repair. HELIYON.

[13] Mocanu I, Van Wettere M, Absil J, Bruneau M, Lubicz B, Sadeghi N (2018). Value of dual-energy CT angiography in patients with treated intracranial aneurysms. Neuroradiology.

[14] Chen Y, Lu C, Chen Y, Chen H, Hsieh T (2022). Reliability and benefits of single-energy projection-based metallic artifact reduction (semar) in the different orthopedic hardware for the hip. SKELETAL RADIOL.

[15] Sonoda A, Nitta N, Ushio N, Nagatani Y, Okumura N, Otani H (2015). Evaluation of the quality of ct images acquired with the single energy metal artifact reduction (semar) algorithm in patients with hip and dental prostheses and aneurysm embolization coils. JPN J RADIOL.

[16] Jabas A, Abello MM, Altmann S, Ringel F, Booz C, Kronfeld A et al. Single-energy metal artifact reduction (semar) in ultra-high-resolution ct angiography of patients with intracranial implants. DIAGNOSTICS 2023;13. 10.3390/diagnostics13040620 PMC9955916 36832109

[17] Pan YN, Chen G, Li AJ, Chen ZQ, Gao X, Huang Y (2019). Reduction of metallic artifacts of the post-treatment intracranial aneurysms: effects of single Energy Metal Artifact Reduction Algorithm. Clin Neuroradiol.

[18] Sarada Prasad D (2015). LV Segmentation using Stochastic Resonance and Evolutionary Cellular Automata. Int J Pattern Recognit Artif Intell.

[19] Sarada Prasad SNIGDHAM (2022). Toward Computing Cross-modality symmetric Non-rigid Medical Image Registration. IEEE Access.

[20] Dakua SP, Abinahed J, Al-Ansari A (2015). Semiautomated hybrid algorithm for estimation of three-dimensional liver surface in CT using dynamic cellular automata and level-sets. J Med Imaging (Bellingham).

[21] Tanabe N, Sakamoto R, Kozawa S, Oguma T, Shima H, Shiraishi Y (2022). Deep learning-based reconstruction of chest ultra-high-resolution computed tomography and quantitative evaluations of smaller airways. RESPIR INVESTIG.

[22] Nakamura Y, Higaki T, Tatsugami F, Honda Y, Narita K, Akagi M (2020). Possibility of deep learning in medical imaging focusing improvement of computed tomography image quality. J COMPUT ASSIST TOMO.

[23] Dabli D, Loisy M, Frandon J, de Oliveira F, Meerun AM, Guiu B (2023). Comparison of image quality of two versions of deep-learning image reconstruction algorithm on a rapid kv-switching ct: a phantom study. EUR RADIOL EXP.

[24] Greffier J, Dabli D, Hamard A, Belaouni A, Akessoul P, Frandon J (2022). Effect of a new deep learning image reconstruction algorithm for abdominal computed tomography imaging on image quality and dose reduction compared with two iterative reconstruction algorithms: a phantom study. QUANT IMAG MED SURG.

[25] Tsuboi K, Osaki N, Ohtani Y, Tanikawa K, Kaneko M (2022). Influence of field of view size and reconstruction methods on single-energy metal artifact reduction: a phantom study. PHYS ENG SCI MED.

[26] Mizuki M, Yasaka K, Miyo R, Ohtake Y, Hamada A, Hosoi R et al. Deep learning reconstruction plus single-energy metal artifact reduction for supra hyoid neck ct in patients with dental metals. CAN ASSOC RADIOL J 2023:719298504. 10.1177/08465371231182904 37387607

[27] Hosoi R, Yasaka K, Mizuki M, Yamaguchi H, Miyo R, Hamada A (2023). Deep learning reconstruction with single-energy metal artifact reduction in pelvic computed tomography for patients with metal hip prostheses. JPN J RADIOL.

[28] Zhang FL, Li RC, Zhang XL, Zhang ZH, Ma L, Ding L (2021). Reduction of metal artifacts from knee tumor prostheses on ct images: value of the single energy metal artifact reduction (semar) algorithm. BMC Cancer.

[29] Shehata MA, Saad AM, Kamel S, Stanietzky N, Roman-Colon AM, Morani AC (2023). Deep-learning ct reconstruction in clinical scans of the abdomen: a systematic review and meta-analysis. ABDOM RADIOL.

[30] Mori M, Fujioka T, Hara M, Katsuta L, Yashima Y, Yamaga E et al. Deep learning-based image quality improvement in digital positron emission tomography for breast cancer. DIAGNOSTICS 2023;13. 10.3390/diagnostics13040794 PMC9955555 36832283

[31] Singh R, Digumarthy SR, Muse VV, Kambadakone AR, Blake MA, Tabari A (2020). Image quality and lesion detection on deep learning reconstruction and iterative reconstruction of submillisievert chest and abdominal ct. AM J ROENTGENOL.

[32] Tamura A, Mukaida E, Ota Y, Nakamura I, Arakita K, Yoshioka K (2022). Deep learning reconstruction allows low-dose imaging while maintaining image quality: comparison of deep learning reconstruction and hybrid iterative reconstruction in contrast-enhanced abdominal ct. QUANT IMAG MED SURG.

