# Supplementary figures and images for: Enhancing image quality in computed tomography angiography follow-ups after endovascular aneurysm repair: a comparative study of reconstruction techniques
Source: BMC Med Imaging. 2024 Jul 1;24:162. doi: 10.1186/s12880-024-01343-z (PMC11218285; doi:10.1186/s12880-024-01343-z)

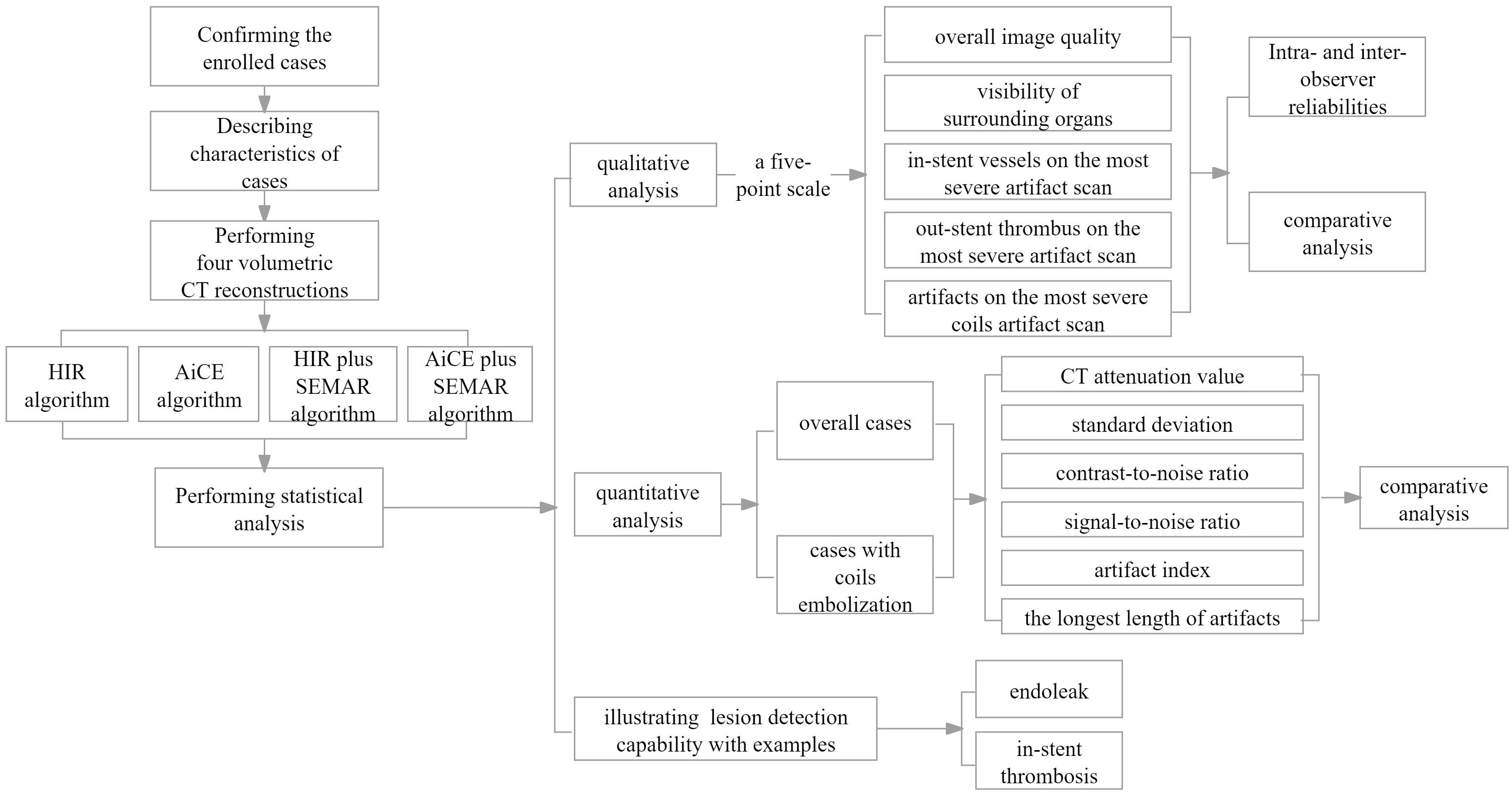

Supplement: Supplementary file 2 — Supplementary Material 2 [file 12880_2024_1343_MOESM2_ESM.jpg]

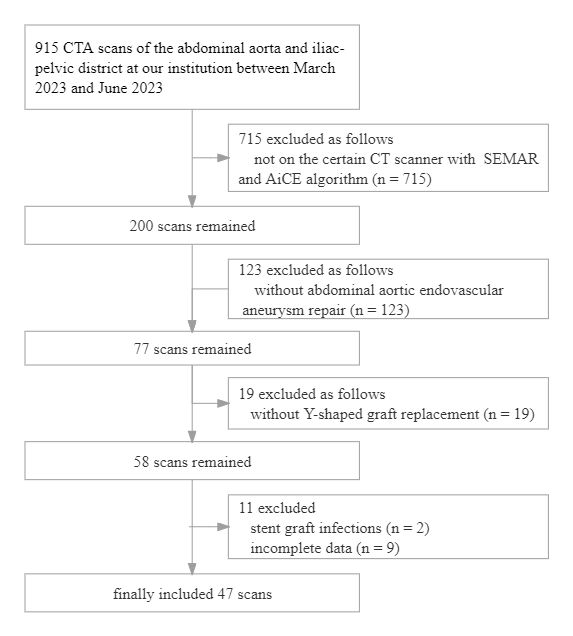

Supplement: Supplementary file 3 — Supplementary Material 3 [file 12880_2024_1343_MOESM3_ESM.jpg]

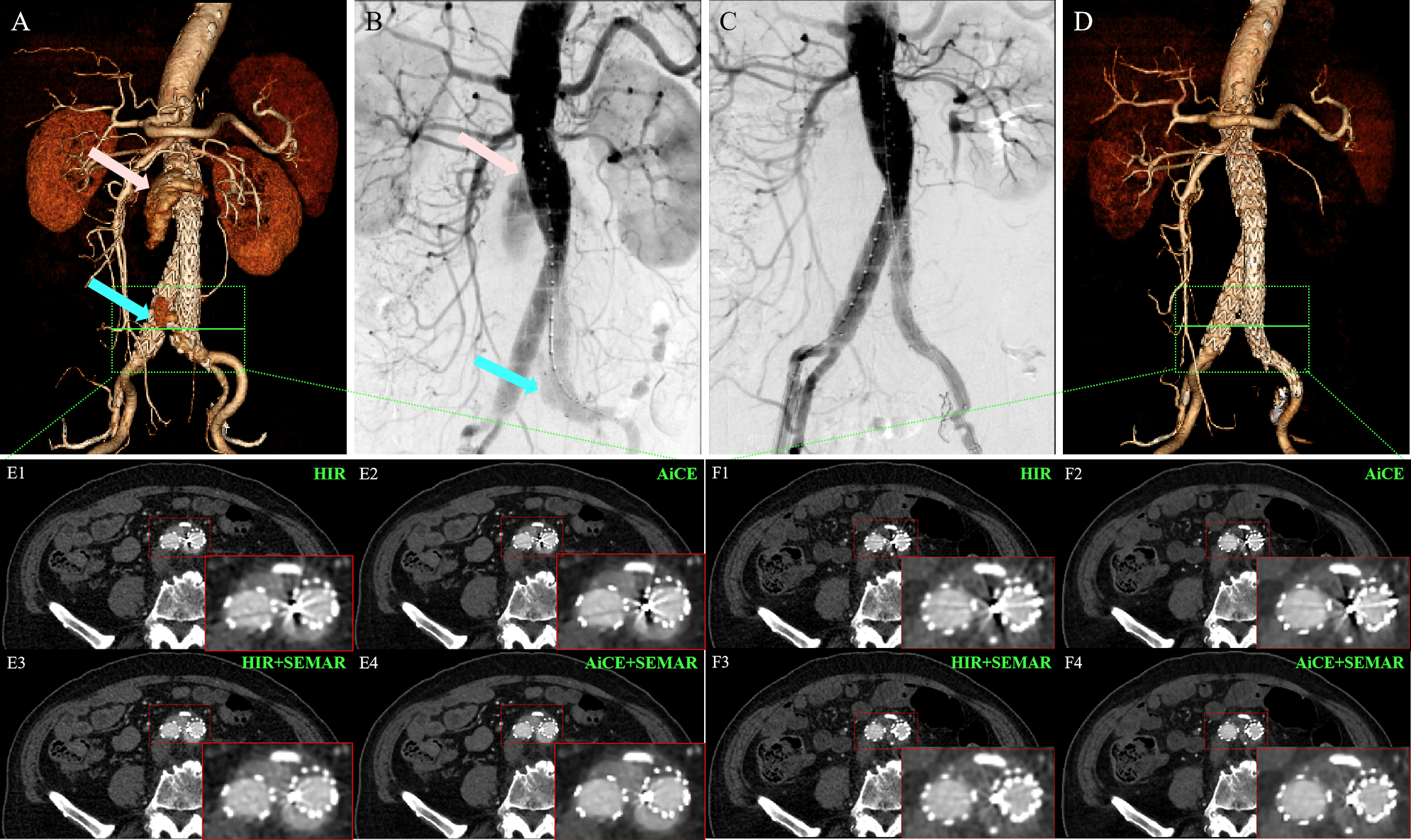

Supplement: Supplementary file 6 — Supplementary Material 6 [file 12880_2024_1343_MOESM6_ESM.tif]
