# Supplementary material for: Enhancing image quality in computed tomography angiography follow-ups after endovascular aneurysm repair: a comparative study of reconstruction techniques
Source: BMC Med Imaging. 2024 Jul 1;24:162. doi: 10.1186/s12880-024-01343-z (PMC11218285; doi:10.1186/s12880-024-01343-z)
Supplement: Supplementary file 7 — Supplementary Material 7 [file 12880_2024_1343_MOESM7_ESM.docx]

**Catalogue**

[Catalogue 1](#_Toc19403)

[Supplemental Appendix 1: Methods 2](#_Toc427)

[Detailed CT Scanning protocol 2](#_Toc24163)

[Statistical analysis 2](#_Toc26974)

[Supplemental Appendix 2: Tables 2](#_Toc20593)

[Supplemental Table 1 Detailed results of one-way ANOVA tests derived from ROIs ranging from ROI1-1 to ROI9 2](#_Toc222)

[Supplemental Table 2 Detailed results of one-way ANOVA tests derived from ROI10, ROI11-1 and ROI11-2 7](#_Toc1433)

[Supplemental Appendix 3: Figures 8](#_Toc8340)

[Supplemental Figure 1 The flowchart outlining the research methodology for this article. 8](#_Toc21133)

[Supplemental Figure 2 Flowchart displaying the inclusion-exclusion procedure 9](#_Toc30871)

[Supplemental Figure 3 Detailed results of quantitative analysis for CT value, SD, CNR and AI. . 10](#_Toc17549)

[Supplemental Figure 4 Detailed results of quantitative analysis for SNR.. . 11](#_Toc4251)

[Supplemental Figure 5 Representative images showing excellent visualization of endoleaks with AiCE+SEMAR. 12](#_Toc7694)

[Supplemental Appendix 4: Videos 13](#_Toc23700)

[Supplemental Video 1 The Digital subtraction angiography examination carried out at our institution verified the existence of endoleaks.. 13](#_Toc31062)

**Supplemental Appendix 1: Methods**

**Detailed CT Scanning protocol**

CT scans were performed with patients in the advanced scanning position of the supine foot, and the scan range extended from the diaphragm to the pubic symphysis. CT scanning characteristics included 120 kV tube voltage, automated tube current modulation, 0.75 s/r gantry rotation speed, 1 mm slice thickness, 0.8 mm reconstruction interval , and 515×512 matrix. A high-pressure syringe containing a non-ionic iodinated contrast agent (Iomeron, Bracco, Italy, 400 mg iodine/mL) was used to accomplish enhanced scanning, intravenously supplied at a rate of 4 mL/s and a dosage volume of 1.2 mL/kg body weight, followed by the intravenous administration of 50 mL of normal saline at the same flow rate. The region of interest (ROI) was located in the beginning portion of the abdominal aorta, and the automatic triggering threshold was set to 180 HU. After an inspiratory breath-hold instruction, scanning commences upon reaching the threshold.

**Statistical analysis**

Poor, moderate, good, and excellent reliabilities corresponded to the following ranges: ICC < 0.50, 0.50 ≤ ICC < 0.75, 0.75 ≤ ICC < 0.90, and ICC ≥ 0.90, respectively.

**Supplemental Appendix 2: Tables**

**Supplemental Table 1** Detailed results of one-way ANOVA tests derived from ROIs ranging from ROI1-1 to ROI9

| parameter | kind (mean±SD) | | | | F | *p* |
| --- | --- | --- | --- | --- | --- | --- |
|  | HIR (n = 47) | AiCE (n = 47) | HIR+SEMAR (n = 47) | AiCE+SEMAR (n = 47) |  |  |
| CT-ROI1-1 | 324.62±68.15 | 313.64±77.91 | 361.74±67.90 | 366.10±69.77 | 6.442 | 0.000** |
| CT-ROI1-2 | 361.58±58.38 | 362.18±58.58 | 361.64±58.51 | 362.28±58.66 | 0.002 | 1 |
| CT-ROI2-1 | 363.05±115.02 | 358.24±111.88 | 364.01±87.50 | 362.54±88.11 | 0.03 | 0.993 |
| CT-ROI2-2 | 348.83±78.46 | 349.57±79.41 | 423.65±540.36 | 350.12±80.02 | 0.831 | 0.478 |
| CT-ROI3-1 | 306.27±125.57 | 304.08±130.04 | 338.93±94.91 | 341.72±94.43 | 1.538 | 0.206 |
| CT-ROI3-2 | 336.31±96.11 | 332.24±101.12 | 336.66±95.48 | 335.26±96.48 | 0.02 | 0.996 |
| CT-ROI4-1 | 10.34±36.60 | 5.68±39.58 | 39.74±19.41 | 41.29±18.33 | 18.497 | 0.000** |
| CT-ROI4-2 | 39.39±11.96 | 38.75±11.74 | 39.33±11.81 | 38.70±11.66 | 0.046 | 0.987 |
| CT-ROI5 | 357.41±55.23 | 352.20±74.11 | 357.39±55.24 | 354.13±62.33 | 0.08 | 0.971 |
| CT-ROI6 | -1194.20±1312.70 | -1003.40±2.24 | -1194.24±1313.20 | -1194.87±1312.59 | 0.332 | 0.802 |
| CT-ROI7 | 50.65±8.43 | 50.62±8.41 | 50.65±8.42 | 50.64±8.40 | 0 | 1 |
| CT-ROI8 | 63.72±13.30 | 63.30±13.43 | 63.75±13.30 | 63.30±13.44 | 0.016 | 0.997 |
| CT-ROI9 | 126.12±37.31 | 126.05±37.22 | 125.49±37.68 | 126.05±37.23 | 0.003 | 1 |
| SD-average | 44.76±8.76 | 42.93±10.61 | 30.34±4.87 | 25.35±6.51 | 66.283 | 0.000** |
| SNR-ROI1-1 | 29.46±7.81 | 70.83±20.58 | 33.20±8.25 | 83.91±21.32 | 137.659 | 0.000** |
| SNR-ROI1-2 | 32.80±7.43 | 81.87±19.29 | 33.12±7.32 | 83.04±19.31 | 179.891 | 0.000** |
| SNR-ROI2-1 | 33.10±12.61 | 81.63±32.51 | 33.31±9.43 | 83.16±25.05 | 78.513 | 0.000** |
| SNR-ROI2-2 | 31.62±8.72 | 78.95±22.51 | 40.33±61.64 | 80.26±22.97 | 24.792 | 0.000** |
| SNR-ROI3-1 | 27.78±12.24 | 68.20±30.89 | 31.06±9.96 | 78.07±25.01 | 67.333 | 0.000** |
| SNR-ROI3-2 | 27.78±12.24 | 68.20±30.89 | 31.06±9.96 | 78.07±25.01 | 67.333 | 0.000** |
| SNR-ROI4-1 | 0.94±3.43 | 1.13±9.41 | 3.68±1.97 | 9.60±4.92 | 23.9 | 0.000** |
| SNR-ROI4-2 | 3.57±1.22 | 8.79±3.20 | 3.60±1.20 | 8.89±3.16 | 74.678 | 0.000** |
| SNR-ROI5 | 32.40±7.03 | 79.62±21.41 | 32.71±6.93 | 80.96±19.06 | 155.357 | 0.000** |
| SNR-ROI6 | -104.84±93.52 | -226.89±37.74 | -113.13±149.37 | -267.27±251.17 | 13.062 | 0.000** |
| SNR-ROI7 | 4.62±1.14 | 11.51±2.97 | 4.66±1.12 | 11.66±2.90 | 152.802 | 0.000** |
| SNR-ROI8 | 5.81±1.59 | 14.40±4.18 | 5.87±1.58 | 14.61±4.22 | 116.808 | 0.000** |
| SNR-ROI9 | 11.41±3.64 | 28.40±9.17 | 11.45±3.65 | 28.78±9.18 | 94.678 | 0.000** |
| CNR-ROI1-1 | 5.27±3.61 | 4.84±3.62 | 9.89±4.60 | 11.59±6.61 | 22.926 | 0.000** |
| CNR-ROI1-2 | 49.21±30.53 | 70.27±73.12 | 49.81±35.99 | 65.33±60.15 | 1.006 | 0.394 |
| CNR-ROI2-1 | 5.17±2.61 | 4.69±2.45 | 8.64±4.19 | 9.46±4.07 | 22.734 | 0.000** |
| CNR-ROI2-2 | 51.30±112.91 | 60.40±76.23 | 50.16±108.04 | 69.95±99.49 | 0.301 | 0.825 |
| CNR-ROI3-1 | 4.11±2.76 | 4.21±3.12 | 9.29±6.04 | 10.74±6.98 | 21.143 | 0.000** |
| CNR-ROI3-2 | 47.13±37.36 | 62.71±84.81 | 48.28±43.25 | 72.28±118.34 | 0.735 | 0.533 |
| CNR-ROI4-1 | 0.82±0.53 | 0.88±0.56 | 1.19±1.20 | 1.17±1.09 | 2.141 | 0.097 |
| CNR-ROI4-2 | 3.76±4.99 | 5.01±4.87 | 3.90±5.37 | 6.04±8.15 | 0.498 | 0.685 |
| CNR-ROI5 | 64.55±71.16 | 117.15±126.27 | 53.61±21.96 | 107.63±97.10 | 3.502 | 0.018* |
| CNR-ROI8 | 1.92±1.39 | 4.00±4.29 | 2.42±2.87 | 3.80±3.83 | 2.482 | 0.065 |
| CNR-ROI9 | 8.78±8.78 | 10.93±11.43 | 9.72±10.01 | 10.87±12.48 | 0.316 | 0.814 |
| AI-ROI1-1 | 98.13±47.35 | 106.66±56.45 | 54.52±27.54 | 49.12±26.10 | 23.111 | 0.000** |
| AI-ROI2-1 | 95.18±34.03 | 106.74±46.84 | 61.75±33.02 | 56.21±31.93 | 20.709 | 0.000** |
| AI-ROI3-1 | 119.67±58.24 | 128.37±75.65 | 56.19±26.47 | 49.55±23.95 | 30.24 | 0.000** |
| AI-ROI4-1 | 84.93±31.11 | 87.64±36.97 | 31.03±19.08 | 29.40±20.38 | 61.304 | 0.000** |
| Note: * *p* < 0.05, ** *p* < 0.01. The unit of CT and SD values is HU (Hounsfield unit). | | | | | | |

**Supplemental Table 2** Detailed results of one-way ANOVA tests derived from ROI10, ROI11-1 and ROI11-2

| parameter | kind (mean±SD) | | | | F | *p* |
| --- | --- | --- | --- | --- | --- | --- |
|  | HIR (n = 25) | AiCE (n = 25) | HIR+SEMAR (n = 25) | AiCE+SEMAR (n = 25) |  |  |
| CT-ROI10 | -87.59±63.07 | -99.74±66.19 | 4.02±41.62 | -0.50±42.51 | 25.743 | 0.000** |
| CT-ROI11-1 | 295.15±157.00 | 293.63±162.28 | 171.92±86.80 | 160.72±93.25 | 8.166 | 0.000** |
| CT-ROI11-2 | 392.46±106.75 | 384.36±102.32 | 324.05±101.64 | 323.42±102.59 | 3.289 | 0.024* |
| SD-ROI10 | 183.35±48.94 | 195.00±53.07 | 56.21±31.10 | 53.24±34.46 | 82.12 | 0.000** |
| SD-ROI11-1 | 128.11±76.85 | 137.43±84.57 | 42.62±21.58 | 40.93±27.26 | 19.45 | 0.000** |
| SD-ROI11-2 | 101.42±63.05 | 105.88±73.15 | 50.35±29.25 | 41.88±26.67 | 10.271 | 0.000** |
| SNR-ROI10 | -8.16±5.80 | -9.30±6.17 | 0.36±3.73 | -0.05±3.79 | 26.573 | 0.000** |
| SNR-ROI11-1 | 28.19±17.97 | 28.08±18.56 | 15.54±7.73 | 14.35±7.85 | 7.376 | 0.000** |
| SNR-ROI11-2 | 36.32±12.68 | 35.53±12.12 | 30.00±11.85 | 29.93±11.89 | 2.027 | 0.115 |
| CNR-ROI10 | 1.15±0.55 | 1.17±0.55 | 1.77±1.07 | 2.01±1.24 | 5.686 | 0.001** |
| CNR-ROI11-1 | 3.72±2.96 | 3.64±3.14 | 5.08±3.85 | 4.90±3.20 | 1.239 | 0.3 |
| CNR-ROI11-2 | 6.88±4.30 | 7.05±5.03 | 13.27±12.13 | 11.85±6.56 | 4.489 | 0.005** |
| AL | 4.81±2.39 | 4.81±2.39 | 1.33±0.61 | 1.33±0.61 | 33.251 | 0.000** |
| AI-ROI10 | 182.20±49.07 | 194.35±53.14 | 51.96±31.92 | 50.48±35.07 | 84.029 | 0.000** |
| AI-ROI11-1 | 125.85±77.95 | 136.07±85.35 | 40.53±22.89 | 42.93±27.97 | 15.849 | 0.000** |
| AI-ROI11-2 | 98.52±64.45 | 104.03±74.16 | 45.76±32.15 | 41.98±27.94 | 8.942 | 0.000** |
| Note: * *p* < 0.05, ** *p* < 0.01. The unit of CT and SD values is HU, and the unit of AL values is cm. | | | | | | |

**Supplemental Appendix 3: Figures**


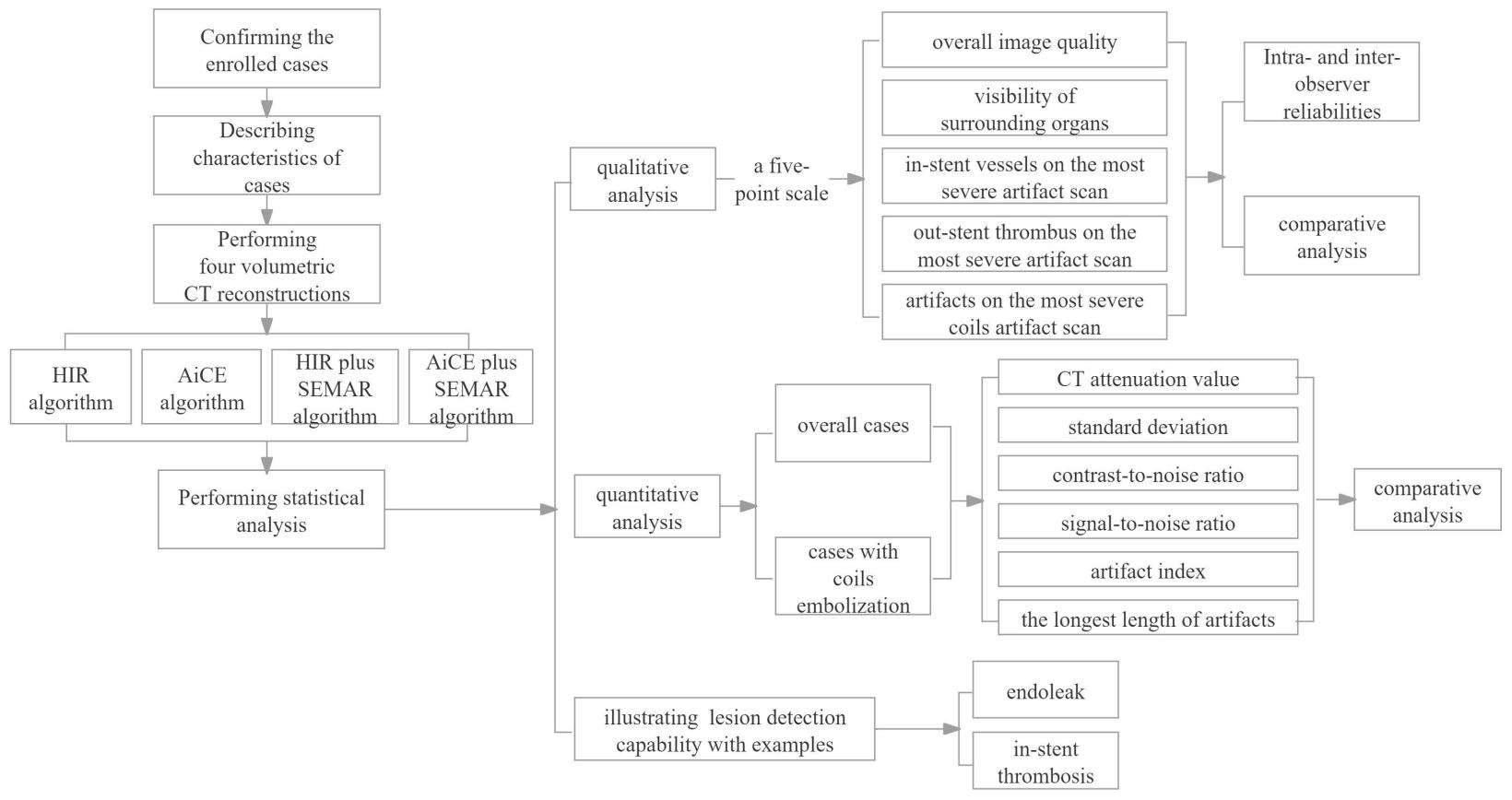


**Supplemental Figure 1** The flowchart outlining the research methodology for this article.

**
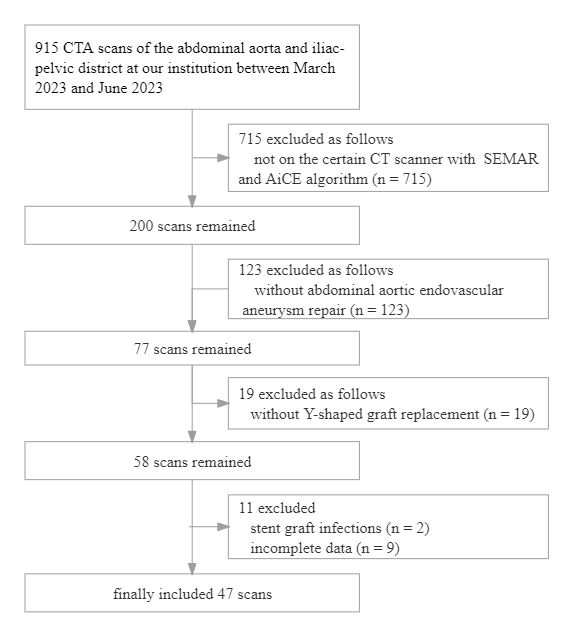
**

**Supplemental Figure 2** Flowchart displaying the inclusion-exclusion procedure and 

showing the final enrolled scan counts.





**Supplemental Figure 3** Detailed results of quantitative analysis for CT value, SD, CNR and AI. **(A-P)** Forest plots displaying results of post hoc pairwise comparisons for CT value, SD, CNR and AI of different ROIs. The ROI designations are in the top left of each image.


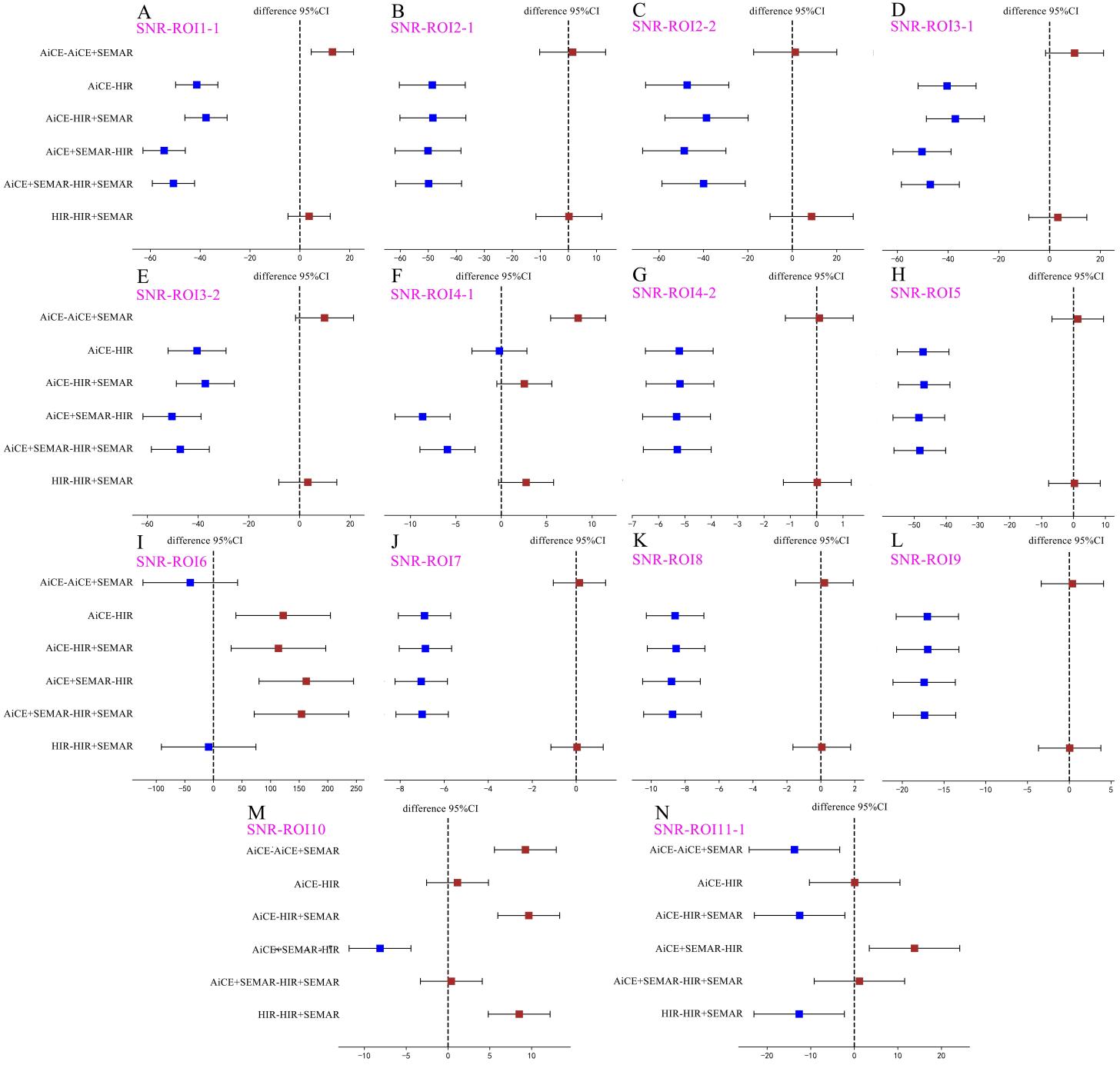


**Supplemental Figure 4** Detailed results of quantitative analysis for SNR. (A-N) Forest plots displaying results of post hoc pairwise comparisons for SNR of different ROIs. The ROI designations are in the top left of each image.


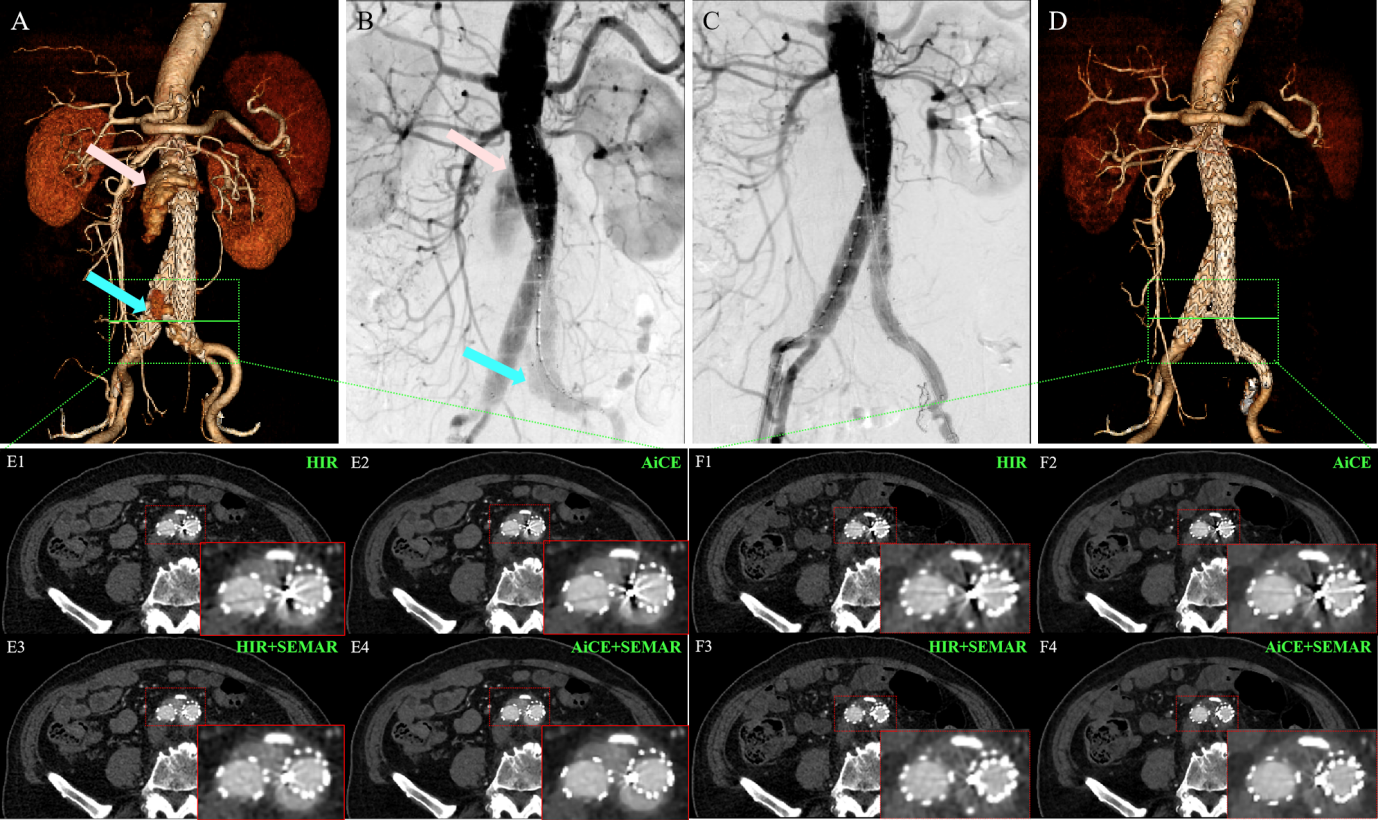


**Supplemental Figure 5** Representative images showing excellent visualization of endoleaks with AiCE+SEMAR. This elderly male patient had undergone abdominal aortic aneurysm endovascular repair seven years ago. Preoperative volume-rendered CTA images (A) and the digital subtraction angiography (DSA) examination (B); postoperative DSA examination (C) and volume-rendered CTA images (D). (pink arrows in A, B) the type Ia endoleak; (cyan arrows in A, B) the type Ib endoleak in the left common iliac artery; (E1-E4) axial CTA examination images employing HIR, AiCE, HIR+SEMAR, and AiCE+SEMAR in turn correlating with the cyan arrows of image A; (F1-F4) postoperative axial CTA examination images corresponding to preoperative images E1-E4. We can see that whether preoperative or postoperative, AiCE+SEMAR imparts images with the best image quality (E4, F4) and provides the most conclusive diagnostic as to whether or not there were endoleaks. The block in the lower left corner of a single image (E1-E4, F1-F4) corresponds to the local amplification of the respective places.

**Supplemental Appendix 4: Videos**

**Supplemental Video 1** The Digital subtraction angiography examination carried out at our institution verified the existence of endoleaks. The patient, a 63-year-old female, had previously undergone endovascular repair for an abdominal aortic aneurysm three months prior.
